# Failure to Thrive Revealing a Pilomyxoid Astrocytoma: An Uncommon Case Report with Literature Review

**DOI:** 10.1155/2021/6670585

**Published:** 2021-09-27

**Authors:** Salma Benyakhlef, Abir Tahri, Asmaa Khlifi, Hajar Abdelouahab, Kamaoui Imane, Fayçal Moufid, Siham Rouf, Hanane Latrech

**Affiliations:** ^1^Department of Endocrinology, Mohammed VI Hospital, Medical School, Mohamed First University, Oujda, Morocco; ^2^Department of Radiology, Mohammed VI Hospital, Medical School, Mohamed First University, Oujda, Morocco; ^3^Department of Neurosurgery, Mohammed VI Hospital, Medical School, Mohamed First University, Oujda, Morocco; ^4^Laboratory of Epidemiology, Clinical Research and Public Health, Faculty of Medicine and Pharmacy of Oujda, Mohamed First University, Oujda, Morocco

## Abstract

Pilomyxoid astrocytoma (PMA) is a freshly described figure of low-grade neoplasms encountered in early childhood. Nevertheless, its precise classification by the World Health Organization (WHO) is still debatable. Making an exact diagnosis relies on histological and immunohistochemical pathognomonic features with specific radiological findings. PMA behaves aggressively with a shorter progression-free survival, and its management is unfortunately still arguable. We describe a rare case of PMA involving the suprasellar region who displays symptoms consistent with diencephalic syndrome. The diagnosis was made by magnetic resonance imaging (MRI) focused on the hypothalamic-pituitary axis, and the patient underwent a subtotal tumor resection combined with chemotherapy. Diagnosis of brain tumors should be kept in mind in young children with generalized and severe unexplained loss of subcutaneous fat with failure to thrive after ruling out classical causes.

## 1. Introduction

Pilomyxoid astrocytoma (PMA) is a rare entity usually described in the hypothalamic-chiasmatic area. A wide variability of diagnostic criteria may overrate the PMA's prevalence by all of the pilocytic neoplasms (PA). It generally concerns infants and very young children (3 months-2 years). Clinical presentation is not well defined. But, the clinicians should be aware of diencephalic syndrome as an unconventional cause of failure to thrive during early childhood which is directly related to neoplasms regarding central nervous system, especially in the hypothalamus and the optic chiasm region. A meticulous assessment of morphologic features and an imminent follow-up is essential because of the aggressivity of PMA and its fluctuating clinical behavior.

We report a case of PMA exhibited by failure to thrive; which is a rare outlined association in the literature.

## 2. Case Report

An 11-month-old boy was admitted to our university hospital for further exploration of poor weight gain (<3rd percentile).

The child was a full-term newborn of first-degree consanguineous parents with an up-to-date vaccination record. The family history was uneventful, and no genetic disorders were reported. Birth growth parameters were within normal ranges, as well as psychomotor development.

At 6 months, the child's weight gain became slow despite increased caloric intake.

**Figure 1:**
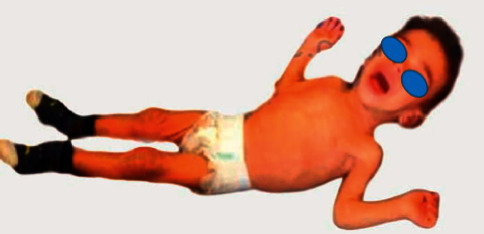
Picture of the child showing loss of subcutaneous fat.

The physical examination revealed pallid and dry skin, besides lack of subcutaneous fat. The patient was afebrile with stable vital signs. His weight was 5 kg with a weight for age value below the third percentile on the grow chart. His head circumference and his length were normal for age. He had a sunken anterior fontanel, a muscle atrophy (Figure 1), and left eye nystagmus. No deficits of movement and coordination were noticed. Deep tendon reflexes were normal as well as cranial nerve function and testing.

Laboratory investigations embracing electrolyte levels, complete blood count, renal function panel, liver parameters, and hormonal assay were normal: FSH (follicle-stimulating hormone): 0.32 mUI/ml (1.5–14), LH (luteinizing hormone): 0.16 mUI/ml (1.2–10), testosterone: 0.078 ng/ml, 8 AM cortisol: 18.5 ug/dl (3.7–19.4), ultrasensitive TSH (ultrasensitive thyroid-stimulating hormone): 2.59 mUI/l (0.27–4.2), and FT4 (free thyroxine): 21.8 pmol/l (12–22). Levels of AFP (alpha-fetoprotein) (0–7) and HCG (human chorionic gonadotropin) (0–7) were also within normal ranges: 1.2 mUI/l and 6.82 ng/ml, respectively. These hormone measurements were based on Chemiluminescence Microparticle Immunoassay (CMIA).

**Figure 2:**
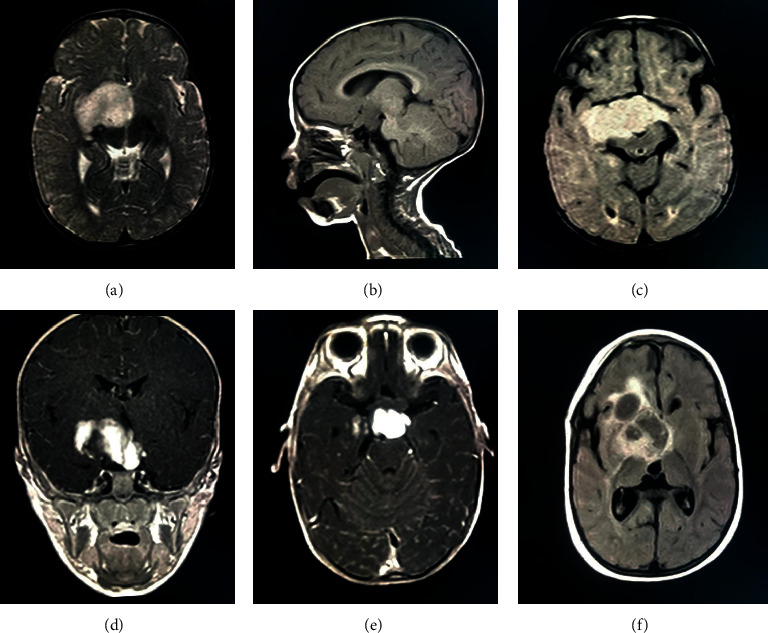
Preoperative axial T2-weighted MRI (a), sagittal T1-weighted MRI (b), and axial FLAIR MRI (c) elucidate a suprasellar lesion isointense on T1-weighted MRI (b) and hyperintense on T2-weighted MRI (a) homogeneously enhanced upon contrast administration (d); 6-month postoperative MRI (e) shows a residual mass with the cystic component (f).

Brain MRI performed upon admission exhibited a suprasellar mass with solid and noncalcified configuration measuring 59∗41∗40 mm in maximum dimensions, without neither peritumoral edema nor parenchymal infiltration. The tumor was isointense on T1-weighted MRI and hyperintense on T2-weighted MRI and homogeneously enhanced upon contrast administration. The lesion extended into the retrosellar region, optic chiasm, and right hypothalamus with a displacement of the anterior and the middle cerebral arteries. The lesion compressed mainly temporal lobes, cerebral peduncles, and the anterior surface of the pons (Figure 2).

The patient underwent a right pterional craniotomy, besides a subtotal tumor resection (95%). Complete excision could not be made because of its anatomical relationships.

After the surgery, the child's clinical course declined with blindness of the left eye and left-sided mild hemiparesis besides focal seizures prevented by levetiracetam twice a day.

Hormonal assessment in the postoperative period showed a panhypopituitarism: TSHus: 0.013 mUI/l (0.64–6.27), FT4: 7.32 pmol/l (12.1–18.6), blood plasma cortisol level at 8 am < 0.2 ug/dl, and ACTH (adenocorticotropic hormone) level <5 pg/ml.

Therefore, hormonal replacement medications have been immediately introduced including hydrocortisone (12.5 mg/day), levothyroxine (25 ug/day), and oral desmopressin (0.2 mg/day).

Histological examination revealed monomorphous bipolar cells with a generous myxoid matrix and an angiocentric disposition of the tumor cells. Rosenthal fibers and eosinophilic granular bodies were not identified, with a very limited number of mitotic figures without any necrotic tissue. Immunochemistry showed oligodendrocyte lineage transcription factor 2 (oligo-2) and S-100 positively stained cells while they seemed negative for IDH1 and P53. The proliferation index of Ki-67 was about 8%.

During a 6-month follow-up, the patient's clinical status was stable since the postoperative phase. MRI of the hypothalamic-pituitary axis showed a residual suprasellar mass with a sellar component compressing the optic chiasm, cerebral pedicles, and pons anterior surface, with the cystic component extending to third and lateral ventricles. Otherwise, a cystic lesion of nearly 17 mm was also noticed. Chemotherapy was suggested, instead of surgical reintervention based on vincristine and carboplatin. The patient died after 2 sessions due to medullary anaplasia.

## 3. Discussion

PMA was first singled out by Tihan et al. in 1999 [1] as a subdivision of astrocytomas.

In 2007, WHO classification [2] denoted it as grade II tumor. However, according to the updated edition of WHO classification of 2016, PMA should not automatically be assigned to this tumoral grade.

In light of recent studies concerning the extensive histology and genetics at the crossroads between PMA and PA, it is suggested to put PMA between brackets until further behavior clarification.

Regardless of thic academic classification, well-conceived cohort studies with considerable evidence showed indisputably that PMA tends to behave more aggressively than PA implying increased mortality rates and a poor disease-free survival [3].

Up to date, PMA incidence statistics remain unknown, but they are rarely reported in the literature (Table 1). However, PAs are the most prevailing primary gliomas during childhood and adolescence accounting for 15.6% of brain tumors [11]. In our country, descriptive cancer data, especially concerning pediatric brain tumors, are not available [12].

These tumors concern mostly young children generally between 3 months and 2 years. American literature mentioned an overall diagnosis mean age of 18 months for PAs [13]. In our case, the patient is 11 months old within the typical age range. PMA can be noticed anywhere in the central nervous system, but the diencephalic region and, especially, the hypothalamic/chiasmatic area are undoubtedly the predilected localizations, as seen in our case.

The clinical manifestations of PMA match with those of other pediatric brain tumors including developmental delay and altered level of consciousness, besides hydrocephalus and generalized weakness along with focal neurological symptoms. Endocrine dysfunction and visual impairment such as nystagmus have been described as a pathognomonic sign in 55% of cases [14].

A rare presentation of hypothalamic/chiasmatic neoplasm is failure to thrive with generalized loss of subcutaneous fat also known as diencephalic syndrome. This syndrome was first described by Russel in 5 children with hypothalamic astrocytoma [15].

In fact, failure to thrive was the presenting symptom in our case, without any defects suggesting chronic malnutrition, allying especially deep emaciation with nystagmus.

The overall uncommoness of this syndrome in this situation causes a considerable time gap between symptom onset and diagnosis.

Failure to thrive takes part of the most frequent troubles in paediatrics. It may be an outcome of diverse disorders such as nervous anorexia; absorption issues (Crohn's or celiac disease …); or any other chronic illness. Subsequently, an uncommon trouble should be suspected if dietary and behavioral interventions have been useless [14]. In fact, brain tumors can be responsible for the diencephalic syndrome including optic and hypothalamic astrocytomas. Nonetheless, approximately 9% of tumors are located elsewhere, including the posterior fossa and anterior hypothalamus [15]. In general, astrocytomas associated with diencephalic syndrome are larger, more aggressive, and occur at a younger age than those described in the absence of this syndrome. Nystagmus, optic atrophy, and tremor were more frequently seen in the anterior fossa group while failure to thrive, hyperactivity, and pallor were equal in both groups [15].

In rare cases, different types of tumors such as ependymomas, dysgerminomas, or gangliocytomas have been associated with diencephalic syndrome. But sometimes, the causative tumor is unclassified [15].

Diencephalic syndrome is rarely described despite its morbidity, which may be responsible for a diagnosis delay for about 11 months from symptoms onset. The initial clinical feature is generally severe emaciation even if the caloric intake is correct [16]. Other symptoms have been illustrated as locomotor hyperactivity, euphoria, and visual disturbances, particularly a nystagmus. The physiological mechanisms are not well explained. In the litterature, different hormones are involved. In fact, high growth hormone concentration and excessive lypolytic peptide secretion (*β*-lipotropin) lead to lipolysis increase and reduction of subcutaneous adipose tissue [16]. Therefore, this syndrome must be kept in mind during childhood, when there is a failure to gain weight with a normal linear growth. Radiological exams have to be performed even without neurological features [16].

MRI imaging (hypothalamic-pituitary MRI imaging) is the gold standard in delineating the central nervous system and seems helpful to identify differential diagnosis and plan the therapeutic strategy. While PA is the most prevalent type of brain tumors in childhood with precise radiographic features, there are few reports describing imaging findings of PMA. It is commonly believed that they can occur anywhere along the neuraxis, but most of them tend to be located in the chiasmatic/hypothalamic region and almost always arise from the midline.

Komotar et al. [16] analyzed 10 PMAs via neuroimaging. The masses are well demarcated without neither infiltration nor peritumoral edema. Solid architecture was observed in 50%, whereas the remaining have some cystic components. T1-, T2-, and diffusion-weighted images showed hyperintense signals in 90%; 89%; and 100%, respectively, of the concerned PMAs. Enhancement pattern was deeply flexible.

Others [17] reported homogeneous enhancement after contrast administration and isointense or sometimes hypointense signal on T1-weighted images besides causing obstructive hydrocephalus or showing rarely central necrosis.

Literature review [18] noticed hemorrhage in 12% opposed to PAs with an estimated rate <1%; that is why it may be the most prominent imaging characteristic evocative of PMA.

Using proton MR spectroscopy [19] demonstrated elevated choline and lipids but decreased creatine and N-acetyl aspartate which are indicative of aggressive tumors and are surprisingly raised in low-grade astrocytomas (PMA and PA).

In our presentation, the MRI showed a suprasellar solid mass measuring 59∗41∗40 mm isointense on T1-weighted MRI, hyperintense on T2-weighted MRI, and homogeneously enhanced after gadolinium injection, extended to the optic chiasm and right hypothalamus.

Obviously, differential diagnosis between PA and PMA seems to be challenging on the ground of imaging findings alone. Histopathological study along with immunohistochemistry of these tumors remains the cornerstone of diagnosis approach and prognosis purposes as well.

The origin of PMA's cell is still ambiguous compared with PA. The bulk of studies proposed an astrocytic origin whereas others lean towards the ependymal or radial glia source [20].

This tumor is identified by monomorphously organized little bipolar cells with spindle-shaped nuclei and a considerable myxoid background. They usually spread from vessels in an angiocentric pattern (pseudorosettes).

PMAs are commonly compact and noninfiltrative, but they can present infiltration and entrap typical brain structures. Rosenthal fibers and eosinophilic granular bodies are missing. Focal necrosis is almost never seen [21].

Immunohistochemical features of PMA are practically close to those of PA. On the one hand, they are positive mainly for GFAP (glial fibrillary acidic protein), S100 protein, vimentin, Olig2 (oligodendrocyte transcription factor), and S0X10 (SRY-related HMG-box). But, on the other hand, they are negative for neuronal markers [21] such as NeuN which is expressed in all glial tumor cell subtypes [19]. Therefore, Ki-67 label index ranges from 1% to 10% in different studies [21].

In our case, histopathology and immunochemistry describe classical features.

The treatment of PMA is still a debatable challenge for every therapist. Moreover, there is no consensus for standard therapy. In fact, surgery or, particularly, gross total resection has an impact on diagnosis, tumor control, and relief of the mass effect over brain elements.

Due to the toxicity of chemotherapy or radiotherapy for infants, adjuvant therapy may be instituted after tumor recurrence following initial gross total resection in case of partial resection with neurological impairment or high Ki-67 index.

In general, chemotherapy is used regardless of the child's age. Nevertheless, radiotherapy is limited to children above 3–5 years of age [22].

The discovery of mTOR and MAPK pathway alterations allowed revolutionary targeted therapy trials [19].

In our case, the surgeon decided to proceed with subtotal tumor resection (95%). Complete excision could not be made because of its anatomical relationships, and chemotherapy was indicated after bringing out a residual mass with the cystic component in 6 months postoperative MRI. This adjuvant therapy was required based on our patient's neurological disorders (blindness of the left eye and left-sided mild hemiparesis besides focal seizures) after the partial tumor excision.

As PMA's clinical experience is not yet rich enough, precising the prognosis of this tumor is a hard labor. As a point of fact, PMA showed a tendancy of aggressivity in comparison with PA. Moreover, a higher rate of local recurrence and dissemination in the cerebrospinal fluid was observed among patients with PMA. Progression-free survival is even shorter than that of PA [23].

Our patient's chemotherapy protocol was designed as follows: an induction period followed by maintenance chemotherapy, for a total of 10 cycles. The assigned chemotherapy medications were vincristine and carboplatin. Unfortunately, our patient died after 2 sessions of chemotherapy due to medullary anaplasia.

According to numerous studies, there is no correlation between BRAF status and the clinical outcome. Nevertheless, others mentioned that KIAA1549-BRAF fusion may improve progression-free survival [21].

Further studies are necessary to identify specific tumor's molecular/genetic abnormalities in order to underline effective therapy and elucidate the patient's outcome.

## 4. Conclusions

The nonspecific clinical aspects of diencephalic syndrome and its fluctuating presentation deeply reflects the lack of understanding of its pathogenesis, and we should keep in mind the necessity of brain MRI within etiological investigations to make diagnosis as early as possible and plan an adequate therapy. We report a rare and neglected presentation of PMA revealed by diencephalic syndrome. That is why every clinician should think of brain neoplasm as a cause of unexplained failure to thrive in childhood.

## Figures and Tables

**Table 1:** Pediatric pilomyxoid astrocytoma described in the recent literature.

Patient	Sex	Age	Anatomic region	Clinical characteristics	Treatment	Follow-up	Author	Year
1 (present case)	M	11 MO	Suprasellar region with local extension	Diencephalic syndrome + nystagmus	Subtotal tumor resection + chemotherapy	Relape after 6 M	(Present case)	2019

2	F	13 MO	Hypothalamic + chiasmatic area	Developmental retardation + nystagmus	Surgery	No relapse during 3-M follow-up	Li et al. [4]	2018

3	M	4 MO	Suprasellar region + right temporal lobe	Vomiting + poor weight gain	Surgery + chemotherapy	Recurrence after 4 Y	Homma et al. [5]	2017

4	F	7 YO	Posterior fossa	Altered consciousness	Surgery	No relapse during follow-up	He et al. [6]	2017

5	F	7 YO	Suprasellar	Intracranial hypertension syndrome	Surgery + radiotherapy	Volume reduction after 12 M	Tjahjadi et al. [7]	2015

6	F	4 YO	Posterior fossa	Ataxia + generalized weakness	Surgery + chemotherapy	Local recurrence after 9 M	Forbes et al. [8]	2013

7	M	6 YO	Suprasellar	Weight loss + developmental delay	Subtotal tumor resection + radiotherapy	No relapse during 3-M follow-up	Azad et al. [9]	2010

8	M	15 YO	Hypothalamic area	Cerebrospinal fluid rhinorrhoea	Surgery + chemotherapy	No recurrence during 18-M follow-up	French et al. [10]	2009

Y = year, M = month, O = old.

## Data Availability

No data were used to support this study.
